# Next-generation sequencing for BCR-ABL1 kinase domain mutation testing in patients with chronic myeloid leukemia: a position paper

**DOI:** 10.1186/s13045-019-0815-5

**Published:** 2019-12-05

**Authors:** Simona Soverini, Elisabetta Abruzzese, Monica Bocchia, Massimiliano Bonifacio, Sara Galimberti, Antonella Gozzini, Alessandra Iurlo, Luigiana Luciano, Patrizia Pregno, Gianantonio Rosti, Giuseppe Saglio, Fabio Stagno, Mario Tiribelli, Paolo Vigneri, Giovanni Barosi, Massimo Breccia

**Affiliations:** 1 Hematology/Oncology “L. e A. Seràgnoli”, Department of Experimental, Diagnostic and Specialty Medicine (DIMES), University of Bologna, S. Orsola-Malpighi Hospital, Via Massarenti 9, 40138 Bologna, Italy; 2 Hematology, S. Eugenio Hospital, ASLRoma2, Rome, Italy; 3 Hematology Unit, Azienda Ospedaliera Universitaria Senese, University of Siena, Siena, Italy; 4 Department of Medicine, Section of Hematology, University of Verona, Verona, Italy; 5 Department of Clinical and Experimental Medicine, Section of Hematology, University of Pisa, Pisa, Italy; 6 Department of Cellular Therapies and Transfusion Medicine, AOU Careggi, Florence, Italy; 7 Hematology Division, Foundation IRCCS Ca’ Granda Ospedale Maggiore Policlinico, University of Milan, Milan, Italy; 8 Hematology Unit, AUOP Federico II, Naples, Italy; 9 Hematology Unit, Azienda Ospedaliero-Universitaria Città della Salute e della Scienza, Turin, Italy; 10 Department of Clinical and Biological Sciences of the University of Turin, Mauriziano Hospital, Turin, Italy; 11 Hematology Section and BMT Unit, Rodolico Hospital, AOU Policlinico-V. Emanuele, Catania, Italy; 12 Division of Hematology and Bone Marrow Transplantation, Department of Medical Area, University of Udine, Udine, Italy; 13 Department of Clinical and Experimental Medicine and Center of Experimental Oncology and Hematology, A.O.U. Policlinico-Vittorio Emanuele, Catania, Italy; 14 Center for the Study of Myelofibrosis, IRCCS Policlinico S. Matteo Foundation, Pavia, Italy; 15 Hematology, Department of Cellular Biotechnologies and Hematology, Sapienza University, Rome, Italy

**Keywords:** Next-generation sequencing, Chronic myeloid leukemia, Sanger sequencing, *BCR-ABL1* mutation

## Abstract

*BCR-ABL1* kinase domain (KD) mutation status is considered to be an important element of clinical decision algorithms for chronic myeloid leukemia (CML) patients who do not achieve an optimal response to tyrosine kinase inhibitors (TKIs). Conventional Sanger sequencing is the method currently recommended to test *BCR-ABL1* KD mutations. However, Sanger sequencing has limited sensitivity and cannot always discriminate between polyclonal and compound mutations. The use of next-generation sequencing (NGS) is increasingly widespread in diagnostic laboratories and represents an attractive alternative. Currently available data on the clinical impact of NGS-based mutational testing in CML patients do not allow recommendations with a high grade of evidence to be prepared. This article reports the results of a group discussion among an ad hoc expert panel with the objective of producing recommendations on the appropriateness of clinical decisions about the indication for NGS, the performance characteristics of NGS platforms, and the therapeutic changes that could be applied based on the use of NGS in CML. Overall, these recommendations might be employed to inform clinicians about the practical use of NGS in CML.

## Background

The introduction of three generations of tyrosine kinase inhibitors (first: imatinib; second: dasatinib, nilotinib, and bosutinib; third: ponatinib) has dramatically changed the management and long-term outcome of patients affected by chronic myeloid leukemia (CML). Nevertheless, resistance has been observed [1–4]. In about one-third of patients who experience resistance to first-line therapy, and in up to 50% of patients who experience resistance to second- or subsequent-line therapy, point mutations in the ABL1 kinase domain (KD) that impair TKI binding can be detected [5]. Mutations may arise at critical contact points between the inhibitor and its target or in key regions of the KD, namely the phosphate-binding loop (P-loop), the catalytic cleft, or the activation loop (A-loop) [5]. One of the most frequent and most challenging mutations is a substitution of threonine with isoleucine at residue 315 (T315I), the so-called “gatekeeper residue” that impairs the binding of imatinib and all second-generation TKIs, and may be overcome only by ponatinib. Detection of a mutation identifies patients at greater risk of subsequent relapse [6]. In the past, mutations (especially the T315I and the P-loop mutations G250E, Q252H, Y253H/F, E255K/V) had also been associated with significantly shorter progression-free and overall survival [7–13]. In more recent times, a negative prognostic impact has not been observed in all studies [14], suggesting that the expanded drug armamentarium and the wider and wider use of mutation testing in routine practice have been beneficial. Indeed, each TKI has its own spectrum of sensitive and insensitive mutations, and the type of mutation detected may, nowadays, drive the choice of TKI to use after failure of first or subsequent lines of treatment (Table 1) [5]. Early identification and quantitative monitoring of mutant *BCR-ABL1* subclones displaying resistance to TKIs are thus important tasks for optimal management of patients with CML.

**Table 1:** List of *BCR-ABL1* KD mutations poorly sensitive to imatinib, dasatinib, nilotinib, bosutinib, and ponatinib based on the integration of published studies (2001–2018) reporting the mutation status of TKI-resistant patients and experimental data

Mutations poorly sensitive to imatinib	M237V, I242T, **M244 V**, K247R, L248V, **G250E**, G250R, Q252R, **Q252H**, **Y253F**, **Y253H**, **E255K**, **E255V**, E258D, W261L, L273M, E275K, E275Q, **D276G**, T277A, E279K, V280A, V289I, V289I, V289A, E292Q, E292V, I293V, L298V, **F311L**, F311I, **T315I**, **F317L**, F317V, F317I, F317C, Y320C, L324Q, Y342H, M343T, A344V, A350V, **M351T**, E355D, **E355G**, E355A, **F359V**, F359I, F359C, F359L, D363Y, L364I, A365V, A366G, L370P, V371A, E373K, V379I, A380T, F382L, **L384M**, **L387F**, L387V, M388L, **H396R**, **H396P**, H396A, A397P, S417F, S417Y, I418S, I418V, A433T, S438C, E450K, E450G, E450A, E450V, E453G, E453A, E453K, E453V, E453Q, **E459K**, E459V, E459G, E459Q, M472I, P480L, **F486S**
Mutations poorly sensitive to dasatinib	V299L, T315I, T315A, F317L, F317V, F317I, F317C
Mutations poorly sensitive to nilotinib	Y253H, E255K, E255V, T315I, F359V, F359I, F359C
Mutations poorly sensitive to bosutinib^a^	E255V, E255K, V299L, T315I
Mutations poorly sensitive to ponatinib	T315M, T315L

^a^In contrast to the other second-generation TKIs, there is still limited data available on mutations associated with clinical resistance to bosutinib in vivo. In vitro data suggest that the E255K and, to a lesser extent, the E255V might be poorly sensitive to bosutinib [15]

*TKI* tyrosine kinase inhibitor

The most frequent imatinib-resistant mutations are highlighted in boldface

Conventional Sanger sequencing is the currently recommended method for diagnostic *BCR-ABL1* KD mutation screening [16]. However, Sanger sequencing has limited sensitivity (it cannot robustly identify mutations present in less than 15–20% of transcripts) and, in many cases, cannot provide clear discrimination between polyclonal and compound mutations [17].

Various next-generation sequencing (NGS)-based approaches facilitating sensitive detection and quantitative monitoring of *BCR-ABL1* KD mutations have recently been described. They are able to detect and quantify any sequence variation in *BCR-ABL1* transcripts down to 1% abundance and resolve the clonal architecture in the majority of cases harboring multiple mutations [18–20]. However, the broad clinical implementation of NGS for *BCR-ABL1* KD mutation testing has so far been hampered by a series of issues, including limited availability due to the high capital investment required to acquire an instrument, and the current costs of the tests, which may not be regarded as readily affordable for routine diagnostic monitoring. Additionally, the currently available data on NGS-based *BCR-ABL1* mutational testing in CML patients who experienced resistance to treatment have not yet been used to formulate recommendations on its utilization in clinical practice.

The objective of this project was to produce consensus-based recommendations on the appropriateness of the clinical decisions concerning the indications of NGS in CML, the performance characteristics of NGS platforms, and the therapeutic changes that could be applied based on the results of NGS analysis.

## Brief overview of NGS methodologies

The term “NGS” refers to different chemistries and platforms that use different strategies to perform the same series of tasks. Briefly, the pool of DNA molecules to be sequenced (usually termed “library”: may be either fragmented genomic DNA or cDNA, or amplicons) need to be physically isolated in space and clonally amplified by polymerase chain reaction. Next, the instrument performs massively parallel sequencing of each individual molecule, yielding millions of sequence reads in a few hours. Key features of NGS are thus the high throughput and the clonal nature of sequence analysis. The high throughput can be exploited to cover whole genomes, or exomes, or transcriptomes in a single sequencing run, or to focus on a panel of genes or genetic regions of interest, that will be sequenced hundreds or thousands of times, thus achieving high sensitivity. The latter currently represents the main diagnostic application of NGS and is frequently referred to as “deep” or ultra-deep sequencing’. For a more detailed technical overview of NGS methodologies, see Yohe S and Thyagarajan B, 2017 and Muzzey D et al., 2015 [21, 22].

## Methods

An Expert Panel (hereafter referred to as the Panel) composed of Italian physicians and biologists was selected for their individual expertise in research and clinical practice in the management of CML and assembled in May 2018. During an initial meeting held the following month, the outline of the project was discussed, and the topics that form the structure of the present document were decided. Key questions were selected through a series of questionnaires, and each panelist drafted statements that addressed one or more questions, while the remaining panelists scored their agreement with those statements and provided suggestions for modifications. Finally, the Panel convened for a consensus conference that was held in Milan, Italy, in November 2018. At this conference, final proposals were given using the nominal group technique [23], by which participants were first asked to comment in a round-robin fashion on their disagreements with the proposed issues and then to vote for a final statement.

## Results

A summary of the recommended indications for the use of NGS testing in CML proposed by the Panel is presented in Table 2.

**Table 2:** Summary of the indications for the use of next-generation sequencing (NGS) for BCR-ABL1 KD mutation testing in chronic myeloid leukemia (CML)

Indications for the use of NGS testing in chronic phase CML	
- in patients with failure^a^ response to TKI therapy, irrespective of the TKI	
- in patients with warning^a^ response to TKI therapy, irrespective of the TKI	
Indications for the use of NGS testing before allogeneic stem cell transplant (allo-SCT)	
- *BCR-ABL1* KD mutation status by NGS testing before allo-SCT may provide useful information regarding when post-transplant TKI therapy should be reinstated. Patients who do not have *BCR-ABL1* KD mutation results by NGS available at the time of transplant should be tested^b^	
Indications for the use of NGS testing in advanced CML phases	
- all patients with advanced phase (AP or BC) either at diagnosis or during therapy	
Indications for the use of NGS testing after TKI therapy discontinuation	
- in patients relapsing after a TFR attempt if they fail to re-achieve MMR within 3–6 months after TKI re-treatment	

^a^It has to be noted that, at present, failure and warning definitions for third-line and beyond are lacking

^b^Provided that *BCR-ABL1* transcript levels are sufficient, i.e., > 0.1%^IS^

*AP* accelerated phase, *BC* blast crisis, *MMR* major molecular response, *TFR* treatment-free remission, *TKI* tyrosine kinase inhibitor, *IS* International Scale

### Indications for the use of NGS testing in chronic-phase (CP) CML

Approximately one-third of CP CML patients with primary or acquired resistance to first or second-generation TKIs harbor mutations in the *BCR-ABL1* KD [6]. Such mutations are not induced by TKIs. The selective pressure exerted by TKIs selects for mutations that occur by chance at resistance-causing residues at any time during therapy. Mutations represent a hallmark of patients at higher risk of acquiring further mutations and of relapse on second- or subsequent-line of therapy [24–26]. No robust evidence has conclusively shown that mutations may already be detectable at diagnosis in CP patients, even when using highly sensitive methods. A recent study combining high-resolution Duplex Sequencing and computational simulations has indeed suggested that, because of the low number of leukemia-initiating cells, CP CML is very unlikely to harbor resistant mutations at the time of diagnosis and, regardless, these would be well below the detection limit of NGS [27].

The detection of *BCR-ABL1* mutations in TKI-treated patients may represent a biological hallmark of disease progression: cells from CP patients harboring mutations have gene expression profiles superimposable to those from blast crisis (BC) patients [28]. In a study in which 319 CP CML patients were routinely monitored for mutations by Sanger sequencing regardless of response status, the identification of mutations even without evidence of imatinib resistance was found to be highly predictive for loss of complete cytogenetic response (CCyR) and progression to advanced phase [8]. NGS was found to anticipate the detection of emerging resistant mutants from 2 to 11 months earlier than Sanger sequencing and, in some instances, could reveal TKI-resistant mutations at the time of major or deeper molecular responses [29]. In another backtracking study, the highly resistant T315I substitution was found to be detectable on average 3 months earlier than with Sanger sequencing [30]. A more recent study performed in an unselected series of 121 CML patients who were systematically screened using NGS, irrespective of their response to first-line TKI therapy, showed that detection of a mutation by NGS was associated with significantly worse outcomes [31]. Patients who developed a KD mutation had a lower cumulative incidence of CCyR and major molecular response (MMR) compared to patients without mutations. Patients with mutations also had worse 5-year progression-free survival and a higher 5-year cumulative incidence of progression compared to patients without mutations [31].

At present, however, both the European LeukemiaNet (ELN) recommendations and the National Comprehensive Cancer Network (NCCN) guidelines anchor KD mutation testing to the lack of optimal response [16, 32, 33]. A more accurate picture of *BCR-ABL1* mutation status at the time of failure, when therapy has to be changed, may help in selecting the most active TKI. In imatinib-resistant patients who failed second-line dasatinib or nilotinib therapy because of the selection of a *BCR-ABL1* KD mutation, the same mutation could retrospectively be tracked by NGS back to the time of imatinib failure [29, 34, 35]. The advantage of a more sensitive approach at the time of switching to second-line TKI therapy in imatinib-resistant patients had already been shown by Parker et al. using a mass spectrometry-based approach of mutation analysis [36, 37]. In the case of a clinical response classified as “warning,” more accurate and frequent monitoring is recommended in order to allow a prompt change in therapy [33]. In this clinical setting, emerging resistant clones could be present below the detection limit of Sanger sequencing and might thus not be identified in a timely manner, ultimately leading to treatment failure. Indeed, in another “backtracking” study performed in 51 CP CML patients who had acquired TKI-resistant mutations on second-line therapy, the first detection by NGS occurred at the time of a “warning” response in many cases [38]. Unfortunately, however, there are currently no formal definitions of failure and warning beyond second-line, although an update of the 2013 ELN recommendations is planned. Patients on second-line therapy may harbor multiple mutations. In these cases, NGS can define whether such mutations are compound or polyclonal, guiding proper clinical management - because compound mutants and, in particular, those including the T315I have been predicted to be highly resistant to all second-generation TKIs [39, 40].

#### Consensus statements

Neither conventional Sanger sequencing nor NGS for *BCR-ABL1* KD mutation testing are indicated in patients with CP CML at diagnosis, before the start of first-line TKI therapy.

*BCR-ABL1* KD mutation testing by NGS is indicated in CP CML after first-line TKI therapy in patients with a “warning” response, regardless of the generation of TKI used for first-line therapy. In these patients, NGS may detect emerging resistant mutants earlier than Sanger sequencing and could allow a timely therapeutic switch, when appropriate.

*BCR-ABL1* KD mutation testing by NGS is indicated in CP CML after first-line TKI therapy in patients with a “failure” response, independently from the generation of TKI used for first-line therapy. In these patients, NGS could detect a complex pattern of mutations, including low-level and/or compound mutations, and could allow more individualized therapeutic decision-making.

*BCR-ABL1* KD mutation testing by NGS is also indicated in case of a “warning” or “failure” response after second-line TKI therapy. In this situation, the identification of mutations not detectable by Sanger sequencing could allow a more appropriate change of therapy.

The panel agreed that, at present, it is not possible to provide recommendations on NGS testing in patients who are receiving third- or later-line TKI therapy due to the lack of definitions of “optimal,” “warning” and “failure” responses in that setting.

### Indications for the use of NGS testing before allogeneic stem cell transplant

Allogeneic stem cell transplant (allo-SCT) is still the only available treatment that is considered as a curative option, although the high procedural morbidity and mortality remain a major deterrent. The current indications for allo-SCT in CP CML are as follows: failure of nilotinib or dasatinib in the first line or failure of two TKIs or evidence of T315I mutation [33]. The majority of patients who underwent an allo-SCT in the European Group for Blood and Marrow Transplantation (EBMT) database were treated in the pre-ponatinib era, limiting the possibility of establishing whether specific settings, such as CP patients with T315I mutation or patients resistant to a frontline second-generation TKI, are in fact real candidates for this procedure. Indeed, allo-SCT is recommended for all BC patients and for accelerated phase (AP) patients who do not achieve an optimal response [33, 41, 42].

The detection of low-level mutations after TKI failure may become important in opting for stem cell transplant procedure in the presence of a T315I mutation or when multiple mutations are present. In the latter case, it will be important to distinguish between compound and polyclonal mutations with or without T315I. Relapse after allo-SCT in CML is observed in 20 to 40% of patients. In this setting, a continued regular long-term longitudinal monitoring of quantitative *BCR-ABL1* transcript levels post-transplant is crucial to anticipate the occasional late-relapsing patients. The detection of *BCR-ABL1* transcripts in the first few months after transplant seems not to be associated with a worse long-term outcome. Furthermore, it has been shown that persistence of very low levels of residual disease (*BCR-ABL1* < 0.1%^IS^) detectable up to 10 years post-transplant has less implication for relapse [43]. It has been suggested that pre-transplant mutation analysis should be considered when selecting a TKI for post-transplant prophylaxis, based on the observation that the majority of resistant mutations are still detectable after transplantation and that patients often relapse with these mutant clones despite receiving TKI therapy [44, 45].

#### Consensus statements

Although the decision to direct a patient to SCT is based on his/her past clinical history of treatment failure, the Panel recognized that the knowledge of pre-transplant mutation status might be useful should TKI treatment be restarted afterward. Thus, cases who did not undergo NGS testing at the last treatment failure before transplant should be analyzed, provided that BCR-ABL1 transcript levels make it feasible (e.g., are > 0.1%^IS^).

In case of disease relapse or failure to achieve an optimal response with the re-administration of TKIs after allo-SCT, the recommended method of assessing *BCR-ABL1* KD mutation status remains Sanger sequencing because no studies have yet investigated the clinical value of low-level mutations in this setting. However, it can reasonably be assumed that low-level mutations may display the same kinetics of selection under TKI pressure. The Panel agreed that the use of NGS testing for *BCR-ABL1* KD mutations in this setting should be investigated.

### Indications for the use of NGS testing in advanced CML phases

Fewer than 5% of patients with CML are diagnosed in advanced disease, as AP or BC. Furthermore, a small proportion of patients (5–6%) diagnosed in CP become resistant and progress to advanced disease during treatment [46, 47]. One of the hallmarks of AP and BC is genetic instability, which fosters the development of additional cytogenetic abnormalities and point mutations, including mutations in the *BCR-ABL1* KD [48–50]. Accordingly, the frequency of *BCR-ABL1* KD mutations has been reported to be much higher in AP/BC patients (70–80%) than in CP patients [51]. Additionally, BC patients frequently harbor multiple mutations whose clonal relationship cannot always be easily established by Sanger sequencing, unless a cumbersome process of cloning and sequencing is undertaken [39]. In BC patients, NGS has shown that different Ph + subpopulations may follow different routes to escape TKI inhibition, so that mutants detectable by Sanger sequencing may coexist with mutants detectable only by NGS [34]. A series of early case reports has also suggested that mutations may be detectable already at the time of diagnosis in patients who present in AP/BC, especially when highly sensitive assays are used [52–54].

#### Consensus statements

In patients with advanced-stage CML at diagnosis or in patients who progress to AP/BC during therapy, the use of NGS testing for *BCR-ABL1* KD mutations could reveal both single mutations, especially T315I, and low-level compound mutations more frequently than Sanger sequencing. The Panel agreed that searching for *BCR-ABL1* KD mutations by NGS testing is indicated to allow for personalized therapy planning.

### Indications for the use of NGS testing after TKI therapy discontinuation

Treatment discontinuation, commonly referred to as “treatment-free remission” (TFR), is an appealing goal of CML therapy. CML patients with a sustained and stable deep molecular response (MR^4^ or greater) for more than 2 years are possible candidates for discontinuation. Since the first prospective TFR trial (the STIM-1 study in 2007), more than 2000 CML patients worldwide have pursued TKI discontinuation in a clinical trial [55]. Reports from these studies (recently reviewed in [56]) have demonstrated that not all patients eligible for TFR will maintain a deep molecular response once TKI therapy has been discontinued. Invariably, 40–60% of patients will lose their molecular response (defined as the loss of MMR) and will have to resume therapy. The long-term follow-up is consistent across several studies, including patients treated with second-generation TKIs. In the majority of patients, relapses occur rapidly within 6 months after treatment discontinuation [56].

Patients in molecular relapse who promptly restart TKI therapy remain responsive to re-treatment and rapidly regain MMR after 2–3 months, and deeper responses thereafter [57]. So far, only one case has been reported to have developed a mutation at the time of molecular relapse [58].

#### Consensus statement

While NGS testing for *BCR-ABL1* KD mutations has no additional value in patients losing deep molecular response after TKI discontinuation, it is indicated in patients relapsing after a TFR attempt if they fail to re-achieve MMR within 3–6 months after TKI re-treatment.

### Impact of NGS on therapeutic decisions

The recent study by Kizilors et al. in an unselected series of patients systematically analyzed by NGS regardless of their response to TKI therapy showed that patients positive for mutations by NGS have significantly worse outcomes in terms of loss of MMR and CCyR and probability of progression [31]. In that study, a threshold of 3% was established based on a thorough methodological validation, which also led to ISO 15189 accreditation of the assay [31]. In the NGS studies in TKI-resistant patients published so far, no low-level mutation known to be resistant to the TKI the patient was receiving failed to undergo clonal selection [29–31, 34, 35, 38]. Some studies even included a control group of patients who responded to therapy or who relapsed with no evidence of mutation selection by Sanger sequencing [30, 35, 38], and in these groups, no low-level mutation resistant to the TKI in use were detected. In all of these studies, the lower detection limit of NGS was set to 1–3% [29–31, 34, 35, 38, 59–62]. In the prospective multicenter study “NEXT-in-CML,” a lower detection limit of 3% was chosen after a control round aimed at testing inter-laboratory reproducibility, showing that in between 1 and 3% of variant frequency, some false positive and false negative mutation calls may occur [63]. Additional studies will be needed to confirm a threshold showing the relevance and robustness at both the technical and clinical levels. In particular, the relevance of low-level mutations for whom inconclusive or no in vitro or in vivo sensitivity data are available is still unknown, and whether they can represent at least a marker of greater genetic instability is under investigation.

#### Consensus statements

In general, the interpretation of the results of *BCR-ABL1* KD mutation testing by NGS and the consequent clinical decisions should involve both biologists and clinicians expert in CML biology and treatment.

The Panel agreed that whether a positive mutation result by NGS testing should lead to an immediate change of treatment depends on the level of non-optimal response to TKIs, the type and level of mutation(s) detected by NGS, and clinical considerations as to the suitability of therapeutic alternatives for each individual patient.

The Panel argued that the greatest utility of *BCR-ABL1* KD mutation testing by NGS is in CP CML patients with a “failure” or “warning” response. In the latter setting, the detection of any mutation known to be associated with resistance to the TKI the patient is receiving at a level > 3% should be strongly considered for a change of therapy; if the mutation level is between 1 and 3%, testing a subsequent sample in 1 month is recommended in order to check for mutation kinetics. An increase in mutation burden should trigger a change of therapy.

### Performance characteristics of NGS testing in CML

TKI-resistant mutations have been reported all over the KD [6]. Mutations outside the KD have been investigated and described in only a single study [64], but their clinical relevance, if any, is unknown. There are currently no European Conformity (CE)-marked for in vitro diagnosis (CE-IVD) or Food and Drug Administration (FDA)-approved commercial kits available for NGS-based *BCR-ABL1* KD mutation testing. The so-called “myeloid panels” include the *ABL1* exons coding for the KD, but DNA-based mutation screening would mainly have untranslocated *ABL1* as a substrate, thus dangerously “diluting” mutations down to a level that might be undetectable even by NGS. Some studies have reported the setup and application of in-house-developed protocols for *BCR-ABL1* KD mutations screening implemented on different NGS platforms [29–31, 34, 35, 38, 60–62].

The results of these studies suggest that NGS-based *BCR-ABL1* KD mutation screening is technically feasible in expert laboratories and may provide accurate and reproducible results even down to variant frequencies of 1%. However, inter-lab reproducibility is equally fundamental for diagnostic accuracy and homogeneity of patient management. National or international expert networks exist that have played, and will continue to play, a key role in the standardization and harmonization of diagnostic tools. For example, at the European level, the “EUTOS (European Treatment and Outcome Study) for CML” initiative, that over the past decade has been running regular control rounds for real-time quantitative polymerase chain reaction (PCR)-based molecular response monitoring, has recently undertaken the first control round of NGS-based *BCR-ABL1* KD mutation screening.

#### Consensus statements

Any NGS-based *BCR-ABL1* KD screening assay should have three mainstays:

a) RNA from peripheral blood buffy coat as a template. Any in-house or commercial method of RNA extraction may be used provided that it ensures at least one microgram of high-quality RNA;

b) Selective analysis of the *ABL1* KD derived from the fusion *BCR-ABL1* allele. This may be accomplished using two alternative forward primers, either on *BCR* exon 1 (for the e1a2 breakpoint; p190) or on exon 13 (for the b2a2 and b3a2 breakpoints; p210) and a common reverse primer on *ABL1* exon 10. The resulting amplicon may either be used as a template for a nested PCR or may be enzymatically fragmented, provided that the resulting amplicons or fragments are of adequate length, as detailed below;

c) Amplicon/fragment length not shorter than 300 base pairs (bp; excluding adapters and indexes). Accordingly, sequencing chemistry and cycles producing reads shorter than 400 bp are not recommended, since this would be of limited value for the detection of compound mutations. The minimal region to be screened for mutations must include an mRNA sequence (reference: Genbank accession no. NM_005157.5) corresponding to amino acids 235 through 498 of the ABL1 1a protein isoform (KD). Mutations outside this region, if detected, should not be reported.

The minimum recommended depth of coverage is 1000×; better if greater. Until commercial kits are available, each individual laboratory will be responsible for the optimization of assay conditions and will have to carefully assess accuracy, precision (repeatability), and analytical sensitivity.

Given the inherent differences between NGS platforms, protocols, and bioinformatics tools, specific recommendations on ranges and thresholds cannot be provided. It is recommended that the performance requirements for each individual assay be established during the validation procedure, and the same procedure is used to monitor the performance of the assay for each run. It is also recommended that each lab engages in regular external quality assurance programs.

Mutations with a variant allele frequency below 1% should not be reported. The exact reportable range, however, should be established and validated by each individual lab. Any variant should be reported, but those for whom experimental or clinical information regarding the sensitivity profile is available should be clearly distinguished from those with an unknown sensitivity/resistance profile, and the TKIs not likely to be effective against those mutants should be indicated, with references to the existing literature.

### Strength of the recommendations on indications of NGS testing

The Panel discussed the issue of how binding clinical centers that do not have access to NGS technology should consider the indications for the use of NGS testing for *BCR-ABL1 *KD mutation issued in this document.

The Panel agreed that NGS testing should be encouraged in the proper indications for use in all the clinical centers that care for patients with CML. However, the Panel also argued that in no CML setting (CP, AP, BC) has the lack of access to NGS testing been demonstrated to hamper the possibility of properly managing CML patients. Thus, failure to perform an NGS test for *BCR-ABL1* KD mutations currently does not represent inappropriate clinical management of patients.

The Panel also suggested that NGS testing for *BCR-ABL1* KD mutations should be performed in a limited number of highly specialized and qualified laboratories within regional or national networks, undergoing periodic and regular quality control rounds.

## Discussion

*BCR-ABL1* KD mutation testing is recommended both by the ELN and by the NCCN in CML patients who do not achieve an optimal response to TKI therapy. While NCCN does not address any methodological aspect, the ELN, in 2011, endorsed Sanger sequencing as the gold standard for *BCR-ABL1* KD mutation screening. The ELN panel acknowledged that the limited sensitivity (15–20%) of Sanger sequencing might be a drawback, but admitted that Sanger sequencing was, at that time, the only method enabling the scanning of the entire KD for the multitude of mutations associated with imatinib and second-generation TKI resistance [16]. At present, however, an increasing number of laboratories are in the process of implementing the NGS technology and integrating NGS results into the diagnostic algorithms of patients with various hematological malignancies. Over the past five years, a series of studies exploring the use of NGS for *BCR-ABL1* mutation testing have assessed its accuracy and reproducibility and have demonstrated that it may provide a more accurate picture of KD mutation status, which may better inform therapeutic decisions [29–31, 34, 35, 38, 60, 62].

In this article, experts in CML judged whether the body of evidence was sufficient to provide recommendations regarding the use of NGS for detecting low-level mutations in the *BCR-ABL1* transcript. Randomized clinical trials assessing the advantage of a change of therapy based on low-level mutations detectable by NGS (as opposed to mutations detectable by Sanger, or as opposed to no mutation testing at all) are lacking. Thus, the advantage of NGS-based treatment decision-making has not yet been formally demonstrated. This forced the Panel to use the method of consensus to shape the recommendations reported in this manuscript, and which are summarized in Table 2.

After careful evaluation of the literature, the Panel recognized that the body of data available supports the use of NGS in case of warning and failure responses to first- or second-line therapy and in case of disease progression from CP to AP or BC. In patients with warning, early detection of an emerging TKI-resistant mutation may play a key role in identifying those patients who might benefit from a proactive TKI switch rather than from a “wait-and-watch” approach. In patients with failure, it has been shown that some cases may harbor mutations below the lower detection limit of Sanger sequencing, and that, even in Sanger sequencing-positive patients, additional low-level mutations relevant for TKI selection may be detectable by NGS. For patients receiving third-line treatment and beyond, there are currently no formal definitions of failure and warning response, hence no published NGS data. Nevertheless, it can be assumed that NGS may equally be useful in patients who do not improve their responses on third-line therapy or beyond, to prevent them from continuing on an inappropriate therapy. In patients who progress to AP or BC, multiple mutations are often detectable [34]; in this setting, NGS may provide a straightforward way to identify compound mutants.

The Panel discussion also highlighted a series of scenarios where very little data are available regarding the frequency and clinical significance of mutations, either detected with Sanger sequencing or NGS. They include the setting of patients who present in AP or BC, the setting of patients who lose MMR after TKI discontinuation or who fail to regain MMR after therapy has been reintroduced, and the post-SCT setting. Further studies are definitely needed in those specific patient populations.

According to the ELN (and the European Society for Medical Oncology [ESMO]) recommendations, detection of a *BCR-ABL1* KD mutation by Sanger sequencing denotes a failure of TKI therapy and mandates a change of the therapeutic strategy [33, 65]. A variety of studies comparing NGS and Sanger sequencing results have shown that NGS and Sanger results are highly concordant for all variants with a frequency of 15% or higher [29–31, 34, 35, 38, 60, 66–72]. The clinical significance of mutation loads between 1 and 15% has been shown by some retrospective studies and, very recently, by a prospective study in which a consecutive series of patients were monitored by NGS irrespective of their response to the ongoing TKI therapy [29–31, 34]. Moreover, a recently completed prospective multicenter study (“NEXT-in-CML”) has shown that low-level, known TKI-resistant mutants identified by NGS remain consistently detectable and increase in burden whenever the TKI they are insensitive to is not changed [63]. In both of these prospective studies, mutations between 1 and 3% were excluded because, in that range, some false positive and false negative results were found to occur. Thus, there are two critical issues around the relevance of low-level mutations: on one hand, what is the “technical” threshold above which a true low-level mutation (and not an amplification or sequencing artifact) can be detected; on the other hand, what is the “clinical” threshold above which a low-level mutation should be incorporated into therapeutic decision algorithms. Both are equally important, and both deserve further investigation. Pragmatically, it is unlikely that a unique threshold of significance can be defined and applied to all mutations and to all patients. While the resistance profile of some mutations is well established, for others there are inconclusive in vitro sensitivity data, or even no data at all. The latter might be passengers rather than drivers and should not trigger an immediate change of therapy in patients not exhibiting a clear “failure” response. Besides this, in each individual case, NGS results should be contextualized and integrated with disease- and patient-specific characteristics and considerations. In this light, and taking into account the uncertainty due to the inherent error rate of the methodology, the Panel agreed that for mutations detected with a frequency between 1 and 3%, validation and monitoring of mutation kinetics in at least one subsequent sample collected in 1 month should be undertaken before taking any decision. In the future, Digital PCR [18] might become the method of choice for rapid and inexpensive orthogonal validation of clinically relevant low-level mutations detected by NGS. However, the existing commercial kits are not suitable for the detection of mutations in BCR-ABL1 transcripts.

Finally, the Panel compiled a series of methodological recommendations, defining the key features and minimal performance characteristics for an NGS-based *BCR-ABL1* KD mutation testing assay. This was felt to be important since no commercial assays are yet available. Thus, each laboratory will be responsible for the design, set up and validation of its own, “home brew” protocol as well as for the regular monitoring of assay performance. For the same reason, the Panel underlined the importance that NGS be performed only by highly qualified laboratories, belonging to specialized national or international networks, ensuring continuous scientific update and cooperating in the standardization of protocols and periodical quality control rounds. Designation of a few expert referral laboratories where samples can be centralized will also be mandatory to ensure reasonable costs and turnaround times.

## Conclusions

Different situations in which NGS may be, to a greater degree than Sanger sequencing, useful in the optimization of the therapeutic strategies have been discussed by a Panel of experts, and a consensus opinion has been reached. With this, we did not mean to compete with or substitute for, but rather to lay the foundations for, the international efforts under the auspices of ELN or other expert networks, that can be predicted to take the lead in the worldwide dissemination of clinical and technical recommendations regarding mutation testing in the near future. Of note, the Panel pointed out that even though encouraged, NGS testing cannot yet be considered mandatory in clinical practice, so that lack of access to NGS cannot be claimed to result in inappropriate patient management. However, the indications for the use of NGS described in this position paper can be expected to improve CML diagnostics by encouraging more widespread use of NGS for detecting *BCR-ABL1* KD mutations while preventing unnecessary and/or poor-quality testing. This will also foster the interaction between molecular pathology centers and provide a stepping-stone towards a common NGS language between different platforms and centers.

## Data Availability

The material supporting the conclusion of this review has been included within the article.

## Abbreviations

allo-SCT: Allogeneic stem cell transplant
AP: Accelerated phase
BC: Blast crisis
CCyR: Complete cytogenetic response
CML: Chronic myeloid leukemia
CP: Chronic phase
EBMT: European Group for Blood and Marrow Transplantation
ELN: European LeukemiaNET
ESMO: European Society for Medical Oncology
EUTOS: European Treatment and Outcome Study
KD: Kinase domain
MMR: Major molecular response
NCCN: National Comprehensive Cancer Network
NGS: Next-generation sequencing
PCR: Polymerase chain reaction
Ph: Philadelphia
TFR: Treatment-free remission
TKI: Tyrosine kinase inhibitor
